# Analysis of Aflatoxins, Fumonisins, Deoxynivalenol, Ochratoxin A, Zearalenone, HT-2, and T-2 Toxins in Animal Feed by LC–MS/MS Using Cleanup with a Multi-Antibody Immunoaffinity Column

**DOI:** 10.1093/jaoacint/qsac035

**Published:** 2022-03-08

**Authors:** Naomi Mackay, Elaine Marley, Dave Leeman, Cezary Poplawski, Carol Donnelly

**Affiliations:** R-Biopharm Rhone Ltd, Block 10, Todd Campus, West of Scotland Science Park, Acre Rd, Glasgow G20 0XA, UK; R-Biopharm Rhone Ltd, Block 10, Todd Campus, West of Scotland Science Park, Acre Rd, Glasgow G20 0XA, UK; R-Biopharm Rhone Ltd, Block 10, Todd Campus, West of Scotland Science Park, Acre Rd, Glasgow G20 0XA, UK; R-Biopharm Rhone Ltd, Block 10, Todd Campus, West of Scotland Science Park, Acre Rd, Glasgow G20 0XA, UK; R-Biopharm Rhone Ltd, Block 10, Todd Campus, West of Scotland Science Park, Acre Rd, Glasgow G20 0XA, UK

## Abstract

**Background:**

Regulations limiting aflatoxin levels in animal feed and guidance values for maximum levels for fumonisins (FB_1_ and FB_2_), deoxynivalenol (DON), ochratoxin A (OTA), zearalenone (ZON), HT-2, and T-2 toxins are in place both to protect animal health and to minimize potential transfer to animal products for human consumption. A multi-mycotoxin method which can handle complex feed matrices such as distillers dried grains with solubles (DDGS) is essential for analysis and accurate quantification without the need to revert to separately analyze individual mycotoxins.

**Objective:**

The objective of this study is to generate single laboratory validation data for a method employing a multi-antibody immunoaffinity column (IAC) capable of providing cleanup for eleven mycotoxins, followed by LC–MS/MS quantification without the need for isotopic labelled and matrix-matched standards. The applicability of method is to be demonstrated for corn feed, pig feed, and DDGS by fortification and naturally occurring mycotoxins covering the range of regulated limits.

**Methods:**

Feed sample (1 kg) ground by milling to approximately 1–2 mm particle size and sub-sample (5 g) extracted with acetonitrile–water–formic acid, passing through a multi-mycotoxin IAC, washing, and eluting prior to LC–MS/MS analysis monitoring selected ion transitions.

**Results:**

Recoveries were in the range 74 to 117% (excluding five outliers) for aflatoxins, FB_1_, FB_2_, DON, OTA, ZON, HT-2, and T2- toxins spiked into three commercial animal feed matrixes (*n *=* *84) and within-day RSDs averaged 1.7 to 10.3% (*n *=* *99).

**Conclusion:**

Single laboratory validation of a multi-antibody IAC method coupled with LC–MS/MS has shown the method to be suitable for accurate quantification of eleven regulated mycotoxins in DDGS, pig feed, and poultry feed.

**Highlights:**

IAC method capable of accurately quantifying eleven regulated mycotoxins in complex feed matrices.

Animal feed has a high potential for contamination with multiple mycotoxins as it is cereal-based with several different ingredients, some particularly prone to, say, aflatoxin contamination and others being sources of *Fusarium* toxins as well as ochratoxin A (OTA; 1–3). Risk assessment of mycotoxins in animal feed takes account not only of potential effects on animal health, but also the risks of transfer of mycotoxins or their metabolites to animal products destined for human consumption (4). Regulatory control of aflatoxin B_1_ (AFB_1_) in animal feed is fairly universal in most jurisdictions primarily in recognition of the need to control the transfer of its aflatoxin M_1_ metabolite into milk (5). Limits for AFB_1_ in the United States range from 20 μg/kg for dairy feed to 300 μg/kg for feed for finishing beef cattle (6). In contrast, the European Union (EU) has a limit for AFB_1_ of 20 μg/kg for complete and complementary feed for cattle, sheep, and goats but 5 μg/kg for complete feed for dairy animals and 10 μg/kg for calves and lambs (7). For other mycotoxins in feed, the U.S. Food and Drug Administration (FDA) and EU have a similar approach with advisory limits or guidance values, respectively, rather than statutory controls. The FDA advisory limits for the sum of fumonisin B_1_ (FB_1_) + fumonisin B_2_ (FB_2_) + fumonisin B_3_ (FB_3_) ranges from 5000 μg/kg for feed for equids and rabbits to 100 000 μg/kg for poultry (6). Guidance values in the EU for the sum of FB_1_ + FB_2_ apply to both the intended feed use and the ingredient itself and are not dissimilar to FDA advisory limits ranging from 5000 μg/kg for feed for pigs and rabbits to 60 000 μg/kg for maize and maize product ingredients (8). Both FDA and EU have advisory/guidance values for deoxynivalenol (DON) the value being dependent on intended use with the lowest values of 1000 and 900 μg/kg respectively applying to pigs, which are the most susceptible animal species. Only the EU has guidance values which extend to OTA, zearalenone (ZON), and the sum of T-2 and HT-2 toxins, the specific levels again depending on the intended use of the feed material (8). The upshot of these limits whether they be for statutory purposes or applied as agreed specifications between feed suppliers and their customers, is that a reliable versatile method is essential which can be applied to all the specified mycotoxins. This method should encompass the wide range of limits and be suitable for matrices ranging from cereals such as corn to complex ingredients such as distillers dried grains with solubles (DDGS). DDGS is a co-product of bio-ethanol production and contains valuable amounts of protein, fat, and fiber and has been used as a feed ingredient in the diets of livestock, poultry, and fish since the 1990s (9). The production of DDGS reached more than 22 million tons in 2019 in the United States indicating its importance to the animal feed sector.

There are a number of published LC–MS/MS multi-mycotoxin methods which have been applied to the analysis of a variety of different animal feed samples. These range from those employing the “dilute-and-shoot” approach of extraction and direct analysis (10–13), through to cleanup by solid-phase extraction (SPE; 14), modified QuEChERS (quick, easy, cheap, effective, rugged, and safe; 9, 15) or employing a multi-toxin immunoaffinity column (IAC; 16, 17). Irrespective of sample preparation and extent of cleanup, all multi-toxin methods have employed LC–MS/MS with multiple reaction monitoring (MRM) for specific detection. These proposed methods have generally reported some single-laboratory method validation performance data, but it is difficult to make direct comparisons of method performance. This is because there are significant differences in the complexity of matrices tested and few have examined naturally contaminated feed samples, relying on fortification. In addition, contamination levels have not always reflected target regulated advisory/guidance levels. In evaluating methods, it is important to recognize the complexity of animal feedstuffs (18) and that methods employing no or minimal sample cleanup may well perform well for relatively “clean” spiked matrices such as wheat or corn, but complex feed materials such as DDGS can present extraction challenges as well as difficult to separate co-extracted interferences (18, 19). Matrix-matched calibration (9, 10), use of stable isotope internal standards (12, 13, 15, 20), and careful selection of LC conditions (20), as well as choosing appropriate ions in MRM (11, 21), have all been employed to at least partially mitigate these effects.

A comparison of IAC, SPE, and QuEChERS approaches to cleanup for LC–MS/MS multi-mycotoxin methods concluded that a modified QuEChERS cleanup gave the best method performance (22). However, the work was only conducted for corn rather than more challenging feed matrices and spiking levels from 2 to 80 μg/kg, whilst realistic for aflatoxins, were very low compared to advisory/guidance levels for *Fusarium* toxins and OTA. The performance of the chosen method in proficiency testing gave consistently low values (despite surprising acceptable *z*-scores). For example, for DON in DDGS the assigned value was 9673 μg/kg but the method gave a result of 4559 μg/kg (47%) and for ZON, with an assigned value of 574 μg/kg, the method gave a result of 231 μg/kg (40%; 22). Matrix-matched calibration using an extract from “blank” feed was employed as with other methods (9, 10) to compensate for background interferences. This approach works well in some areas where blank matrices are easily obtained, but this is not the case for animal feed which is invariably contaminated with several mycotoxins. The requirement to do matrix-matched calibration is therefore onerous especially if a diverse range of feed samples is to be analyzed.

Of the various published multi-mycotoxin LC–MS/MS methods for feed, only one to date has been subjected to an interlaboratory collaborative study (20). This European Commission for Standardization (CEN) standard adopted as an EU official method involved acetonitrile and formic acid extraction, centrifugation, and addition of stable isotope internal standards prior to direct LC–MS/MS determination. Six feed samples were analyzed comprising mixtures of compound feed and cereals, but unfortunately no details were provided to enable the complexity of the feed to be assessed. The use of stable isotope internal standards (12, 13, 15, 20) does avoid matrix-matched calibration, but adds cost to the method as the standards are moderately expensive. Generally, there are far fewer published applications of multi-mycotoxin IAC cleanup for feed analysis which has the attraction of almost universal applicability to a diverse range of feed materials and does not necessitate matrix-matched calibration, stable isotope standards nor optimization of MRM to ensure interferences are avoided. This contrasts with the widespread adoption of IAC columns for individual mycotoxins which are used in EU official methods for aflatoxins, DON, ZON, fumonisins and OTA (23).

A method using a commercial multi-mycotoxin IAC has previously been validated (16, 17) for analysis of 12 mycotoxins in samples of corn, durum wheat, corn flakes, and corn crackers. When assessed by another laboratory (22) recoveries and RSDs were poorer albeit for different spiked matrices and these authors advocated modified QuEChERS as the preferred method (22). Others however have successfully employed the multi-mycotoxin IAC for analysis of various cereals and foodstuffs (24, 25), but have not extensively tested the method for analysis of complex feed samples. In contrast another comparison of IAC, SPE, and QuEChERS (26) found that multi-mycotoxin IACs performed best of the three approaches, although the IACs from two different manufacturers were different from those previously employed in other studies (16, 17).

In this paper we report the results of a single-laboratory method validation of a multi-mycotoxin IAC, but from a different manufacturer to that previously described in the literature (16, 17). This is important as the specifications and performance of IACs depend on the quality of the monoclonal antibodies which differ between different suppliers. We have focused exclusively on method validation using naturally contaminated commercial animal feed samples, obtaining recovery data by spiking for those mycotoxin/matrix combinations where background levels were low or not detectable. The method has been validated with a view to demonstrating applicability for the 11 mycotoxins which are either regulated or for which guidance/advisory limits apply in animal feed.

## Experimental

### Apparatus

- *LC-MS/MS system.—*Sciex QTRAP 4500 tandem quadrupole mass spectrometer coupled to an ExionLC™ AC LC system comprising a quaternary pump, cooled autosampler and heated column oven, all controlled by Analyst software and data processing by Sciex OS software (AB Sciex UK, Ltd, Macclesfield, UK)
- *LC column.*—150 x 3 mm Gemini C-18, 110 Å, 5 μm column (Phenomenex, Macclesfield, Cheshire, UK)
- *Immunoaffinity column.*—Multi-mycotoxin IACs (11+Myco MS-PREP^®^) in wide-body format containing monoclonal antibodies specific to aflatoxins, OTA, fumonisins, DON, ZON, T-2, and HT-2 toxins were obtained from R-Biopharm Rhone Ltd (Glasgow, UK). The columns have a minimum capacity of 175 ng of total aflatoxins, 350 ng of OTA, 4500 ng of FB_1_ and FB_2_ (2:1), 700 ng DON, 650 ng ZON, 350 ng T-2, and 350 ng HT-2 toxins. Recoveries were not less than 80% for aflatoxins (AF), fumonisins, DON, ZON, T-2, and HT-2 when 20 ng of total aflatoxins (AFB_1_ +AFB_2_ + AFG_1_ + AFG_2_, 1:1:1:1), 2000 ng of total fumonisin (FB_1_+ FB_2_, 2:1), 160 ng of DON, 30 ng of ZON, and 120 ng of T-2 and HT-2 toxins (1:1) were applied in 10 mL methanol (MeOH)–phosphate-buffered saline (PBS) (2.8 + 97.2) and not less than 60% for OTA when 5 ng was applied in 10 mL MeOH–PBS (2.8 + 97.2).

### Reagents

Deionized water was obtained using a Milli-Q (Merck Millipore, Massachusetts, USA) laboratory water purification system. LC grade MeOH, LC grade acetonitrile (ACN), ammonium acetate and formic acid (HCOOH) were obtained from Thermo Fisher Scientific (Loughborough, UK). Tween 20 was obtained from Sigma-Aldrich Ltd (Gillingham, UK). PBS tablets were obtained from R-Biopharm Rhone Ltd. One tablet was dissolved in 100 mL water to give an 8.0 g/L solution of sodium chloride, 0.2 g/L potassium chloride, 1.15 g/L di-sodium hydrogen phosphate, and 0.2 g/L potassium dihydrogen phosphate with a of pH 7.3 ± 0.2 at 25°C.

### Reagent Solutions

- *Mobile phase A*.—1 mM ammonium formate and water–methanol–formic acid (94.9 + 5 + 0.1)
- *Mobile phase B*.—1 mM ammonium formate and water–methanol–formic acid (1.9 + 98 + 0.1)
- *Extraction solvent*.—A mixture of ACN–H_2_O–HCOOH (79 + 20 +1).

### Chemical Standards

Aflatoxins and OTA powders were purchased from Sigma-Aldrich Ltd in 5 mg (AFB_1_, AFB_2_, and OTA) and 1 mg quantities (AFG_1_ and AFG_2_) of which the aflatoxins were dissolved in ACN, and OTA in MeOH to give solutions of 1 mg/mL. Similarly, DON and ZON were purchased from Sigma-Aldrich Ltd in 1 mg and 10 mg quantities being dissolved in ACN to give 1 mg/mL and 5 mg/mL solutions, respectively. T2-toxin (5 mg) was purchased from LGC (Teddington, UK) and HT-2 toxin (5 mg) from Sigma-Aldrich Ltd and both were dissolved in ACN to 1 mg/mL. FB_1_ and FB_2_ both 100 μg/mL in ACN–H_2_O (1 + 1) were supplied as individually prepared solutions from Trilogy Analytical Laboratory (Washington, MO) with certified concentrations.

### Preparation of Calibration Standard Solutions

- *Individual AFB_1_, AFB_2_ AFG_1_* *,*  *and AFG_2_ standards (1 μg/mL).—*50 μL 1 mg/mL individual aflatoxin standard was mixed with 50 mL ACN (after removing 50 μL to waste) to give a solution with concentration 10 μg/mL individual aflatoxin. Individual aflatoxin standards (1 μg/mL) were prepared by diluting each 10 μg/mL individual aflatoxin standard 10-fold with acetonitrile.
- *Total aflatoxin standard (1 μg/mL).—*175 mL of each individual AFB_1_, AFB_2_ AFG_1_ and AFG_2_ standard (1 μg/mL) was mixed to give a solution with concentration 1 μg/mL total aflatoxin. Total aflatoxin standards (0.1, 0.01 and 0.001 μg/mL) were prepared by serial 10-fold dilution with MeOH.
- *Ochratoxin standard (1 μg/mL).—*1.5 mL of 1 mg/mL ochratoxin standard was mixed with 15 mL of MeOH (after removing 1.5 mL to waste) to give a solution with concentration 100 μg/mL ochratoxin. Ochratoxin standard (1 μg/mL) was prepared by diluting 100-fold with MeOH. OTA standards (0.1 and 0.01 μg/mL) were prepared by serial 10-fold dilution with MeOH.
- *Total fumonisin standard (100 μg/mL).—*7 mL of 100 μg/mL FB_1_ and 3.5 mL of 100 μg/mL FB_2_ standards were mixed. Total fumonisin standards (10 and 1 μg/mL) were prepared by serial 10-fold dilution with MeOH.
- *DON standard (100 μg/mL).—*466 μL 1.073 mg/mL DON standard was mixed with 5 mL ACNμ (after removing 466 μL to waste) to give a solution with concentration 100 μg/mL DON. DON standards (10, 1, and 0.1 μg/mL) were prepared by serial 10-fold dilution with MeOH.
- *ZON standard (50 μg/mL).—*29.5 μL 5.08 mg/mL ZON standard was mixed with 3 mL ACN (after removing 29.5 μL to waste) to give a solution of 50 μg/mL ZON. ZON standards (5, 0.5, 0.05, and 0.005 μg/mL) were prepared by serial 10-fold dilution with MeOH.
- *Total T-2/HT-2 toxin standard (40 μg/mL).—*80 μL 1 mg/mL T-2 standard and 80 μL 1 mg/mL HT-2 standard were added to 4 mL ACN (after removing 160 μL to waste) to give a concentration of 40 μg/mL total T-2/HT-2 toxins. Total T-2/HT-2 standards (4, 0.4, and 0.04 μg/mL) were prepared by serial 10-fold dilution with MeOH.
- As indicated in Table 1 appropriate volumes of each standard solution were added to 5 mL MeOH–H_2_O (1 + 1, by volume) from which 585 μL had been removed to give “Cal 7 solution” containing 8 ng/mL total aflatoxins, 20 ng/mL OTA, 2000 ng/mL total FB_1_ + FB_2_, 360 ng/mL DON, 40 ng/mL ZON, and 100 ng/mL total T-2 + HT-2 toxins. As indicated in Table 2, sequential dilution of Cal 7 solution was made with MeOH–H_2_O (1 + 1, by volume). Thus, 1 mL Cal 7 solution was mixed with 1 mL MeOH–H_2_O (1 + 1, by volume) to produce Cal 6 which was similarly diluted and so forth to produce Cal 5 to Cal 1.

**Table 1.** Preparation of intermediate solvent calibration solutions

Mycotoxins	Std.^a^ soln.^b^ concn^c^, µg/mL	Vol.^d^ std. soln. (µL) added to 5 mL	Final concn of Cal.^e^ 7, ng/mL
Total aflatoxins	1.0	40	8
OTA	1.0	100	20
FB_1_ + FB_2_	100	100	2000
DON	10.0	180	360
ZON	5.0	40	40
T-2 + HT-2	4.0	125	100

a std. = Standard.

b soln. = Solution.

c concn. = Concentration.

d vol. = Volume.

e Cal. = Calibration.

**Table 2.** Preparation of calibration series by dilution of Cal 7

Cal No.	Calibrant vol, mL	Diluent^a^vol, mL	Concentrations of mycotoxins in ng/mL										
			AFB_1_	AFB_2_	AFG_1_	AFG_2_	OTA	DON	FB_1_	FB_2_	HT-2	T-2	ZON
6	1 Cal 7	1	1	1	1	1	10	180	666.7	333.3	25	25	20
5	1 Cal 6	1	0.5	0.5	0.5	0.5	5	90	333.3	166.7	12.5	12.5	10
4	1 Cal 5	1	0.25	0.25	0.25	0.25	2.5	45	166.7	83.3	6.25	6.25	5
3	1 Cal 4	1	0.125	0.125	0.125	0.125	1.25	22.5	83.3	41.7	3.125	3.125	2.5
2	1 Cal 3	1	0.063	0.063	0.063	0.063	0.625	11.25	41.7	20.8	1.563	1.563	1.25
1	1 Cal 2	1	0.031	0.031	0.031	0.031	0.313	5.625	20.8	10.4	0.781	0.781	0.625

a Diluent MeOH–H_2_O (1 + 1).

### Animal Feed Samples

A naturally contaminated sample of DDGS (MT-DG-9949), which had been homogenized and passed through a 30 mesh (0.595 mm) sieve to ensure homogeneity, was obtained from Trilogy Analytical Laboratory. A poultry feed sample comprising wheat, maize, expelled soya oil, peas, added calcium, and grit intended as a complete feed for laying birds was obtained from a UK feed supplier. Sow and weaner pellets (pig feed) containing wheat, wheat feed, and malt culms intended for sows and piglets was also obtained from a UK feed supplier. A cereal-based animal feed sample (product code FCMA2-AFE2QC; material T04425QC) with assigned values for aflatoxins was surplus FAPAS^®^ test materials purchased from FERA Science Ltd (Sand Hutton, UK). Assigned values for DON, T-2, and HT-2 toxins and ZON in a pig feed sample were obtained through participation in FAPAS Food Chemistry Proficiency Test 22175.

### Spiking of Feed Samples for Recovery Experiments

To achieve the different desired low, medium, and high spiking levels for each mycotoxin in each of the three different feed samples, different volumes of standard solutions were pipetted onto dry feed sample (5 g) pre-weighed into a polypropylene centrifuge tube. The concentrations of the standard solutions employed are shown in Table 1 and the exact volumes of standard solutions used for spiking each feed/mycotoxin combination are shown in the online Supplemental Tables (S1–S3). Before adding the extraction solution, the spiked matrix was allowed to stand for a minimum of 30 min to allow solvent evaporation and to an extent mimic natural contamination by allowing possible mycotoxin-matrix binding to occur.

### Extraction and Cleanup Procedure

Approximately 1 kg feed sample was ground using a Retsch (Haan, Germany) knife mill (GRINDOMIX GM 200) to about 1–2 mm particle size. A test portion (5 g) was weighed into a 50 mL polypropylene centrifuge tube to which 20 mL ACN–H_2_O–HCOOH (79 + 20 + 1) was added and shaken for 30 min. The analyte extract was centrifuged at 4000 reveloutions per minute (rpm) for 10 min. An aliquot of the filtrate (3 mL) was diluted with 147 mL PBS. Twenty milliliters (mL) of the diluted filtrate (equivalent to 0.1 g sample) was passed through the multi-mycotoxin IAC at a flow rate of 2 mL/min. The column was washed by passing 10 mL Tween 20 (0.1%, w/v) in PBS followed by 10 mL 20 mM ammonium acetate at a flow rate of approximately 5 mL/min. Air was passed through the column to remove any residual liquid. Finally, the toxins were eluted from the column at a flow rate of 1 drop per second using 1 mL 100% MeOH and collected in an amber glass vial. The IAC was backflushed by gently raising and lowering the syringe plunger on the glass barrel connected to the IAC during passage of the solvent through the column. This reversed the direction of flow of eluate through the gel and was repeated three times before collecting the eluate. Alternative ways to elute the toxins if preferred are detailed in the suppliers instructions for the multi-toxin IAC (27). Following elution 1 mL H_2_O was passed through the column and collected in the same vial to give a 2 mL total volume. This extract (25 μL) was injected into the LC–MS/MS.

### LC–MS/MS Conditions

- *LC*.—The column temperature was held at 40.0°C. Analyte extracts and solvent standards were placed in an autosampler kept at ambient temperature. The injection volume was 25 μL. The HPLC gradient elution is shown in Table 3. Mobile phase B started at 20% for the first 0.1 min then ramped to 90% over the next 10 min and was held at 90% for 5 min. It was then decreased to 20% over 0.1 min and held at 20% (with 80% A) for 5 min, before the next injection. The LC flow rate was 0.3 mL/min.
- *MS*.—MRM analyses were carried out using electrospray ionization in positive-ion mode. The source temperature was 450°C, ion spray set at 3500 V with ion source gas 1 and 2 (nitrogen) set at 50 and 55 psi, respectively, and curtain gas (nitrogen) operated at 50 psi. Collision energy, cone voltage, and dwell time for each transition were optimized as shown in Table 4. Two transitions for 20 ms (except DON for 100 ms) were monitored for each mycotoxin throughout the entire 20 min chromatographic run time.

**Table 3.** HPLC gradient elution

Time, min	Mobile phase A, %	Mobile phase B, %
0.0	80	20
0.1	80	20
10.0	10	90
15.0	10	90
15.1	80	20
20.0	80	20

**Table 4.** LC–MS/MS conditions for the detection of mycotoxins by MRM

Toxin	RT, min	Precursor ion, *m/z*	Product ions, *m/z*	Dwell time, ms	Collision energy, V	Cell exit potential, V
DON	5.7	297.1	249.0	100	15.0	16.0
			231.0		17.0	15.0
AFG_2_	8.7	331.1	256.9	20	40.9	20.0
			189.1		54.4	14.0
AFG_1_	9.1	329.1	243.0	20	36.6	15.0
			199.9		53.7	15.0
AFB_2_	9.5	315.2	259.0	20	34.9	17.0
			287.1		38.6	17.0
FB_1_	9.5	722.4	334.3	20	55.1	16.0
			352.3		48.3	20.0
AFB_1_	9.8	313.2	285.1	20	30.7	17.0
			241.0		48.2	17.0
FB_2_	10.7	706.4	336.2	20	49.3	20.0
			318.2		50.8	20.0
HT-2	11.1	442.3	263.0	20	16.9	15.0
			215.0		17.2	16.0
T-2	11.9	484.3	305.1	20	17.9	15.0
			245.0		17.3	16.0
ZON	12.8	319.1	283.0	20	16.4	17.0
			187.0		25.9	12.0
OTA	13.0	404.1	239.0	20	31.2	15.0
			358.0		19.1	18.0

### Quantification

Analyst software and data processing with Sciex OS software was used to integrate peak areas of ion chromatograms and calculate the results. For each analyte, the MS/MS transition with the highest ion count was used for quantitation. Seven-point calibration lines for all mycotoxin standards were prepared in solvent with concentrations as shown in Table 2. Linear least-squares regression was applied to construct calibration curves and a high correlation coefficient (>0.99) was required as a criterion of linearity. Concentrations of individual mycotoxins in the sample extracts were calculated using the calibration curves. A method dilution factor of 20 was used to convert results from ng/mL in final extract to the equivalent μg/kg concentration in dry sample.

### Mycotoxin Identification Criteria

Two criteria were employed as confirmatory measures for identification of the respective mycotoxins. Firstly, the chromatographic retention time of the individual mycotoxins should correspond to that of the standards with a tolerance of ±2.5% (28). Secondly, the relative intensities of the detected ions, expressed as a percentage of the intensity of the most intense ion transition, should correspond to the calibration standard, either from calibration standard solutions or from spiked samples, at comparable concentrations, measured under the same conditions, within 20–50% depending on the relative intensity of the ion transition (28).

### Method Validation

- *Spiking*  *procedure.—*The validation was designed to ensure that the method was tested to cover the range of concentrations in the EU regulatory limits for AFB_1_ (7) and the range of guidance values for the other seven mycotoxins of concern (8). For AFB_1_ the lowest regulated level is 5 μg/kg for dairy feed and the highest level is 20 μg/kg for complete feed for pigs and poultry (7) and thus spiking ranged from 2.5 to 20 μg/kg (10 to 80 μg/kg for total aflatoxins). For DON the lowest guidance level is 900 μg/kg for pigs and the highest level is 12 000 μg/kg in maize products and 5000 μg/kg in complementary and complete feed (8) and thus a range of 450 to 5000 μg/kg was selected. For OTA the lowest guidance level is 50 μg/kg for pigs and the highest level is 100 μg/kg for poultry (8) and thus a range of 25 to 100 μg/kg was selected. For ZON, the lowest guidance level is 100 μg/kg for pigs and the highest level is 3000 μg/kg for maize products and 500 μg/kg for dairy cattle and sheep (8) and thus a range of 50 to 500 μg/kg was selected. For the sum of FB1+FB2 the lowest guidance level is 5000 μg/kg for pigs and the highest is 60 000 μg/kg for maize products and 50 000 μg/kg for adult ruminants (8) and thus a range of 2500 to 10 000 μg/kg was selected for total fumonisins, the concentrations being in the ratio 2:1 for FB1:FB2. As maize products are ingredients, the highest permitted levels are not relevant for the finished feed and were not included in the range of levels. For the sum of T2 toxin + HT-2 the lowest guidance level is 250 μg/kg for compound feed and the highest is 2000 μg/kg for oat milling products (8) and thus a range of 125 to 500 μg/kg was selected in equal concentrations.
  The DDGS, poultry feed, and pig feed samples were initially analyzed to determine background levels and where significant contamination was detectable, spiking was confined to those mycotoxins present in not detectable or negligible amounts with respect to guidance values. For the method validation all three samples were found to contain natural contamination with some mycotoxins but also the absence of others. The three feed samples were therefore complementary to one another, thus being suitable for generation of recovery data from spiking as well as precision data from natural contamination.
- *Within-day measurements.—*For within-day experiments to establish recoveries and RSDs at three levels and for the three feed samples all measurements (*n* = 6) were made on a single 5 g feed test portion from which 3 mL extract (from 20 mL) was taken prior to dilution with PBS and IAC cleanup. Each replicate was therefore based on 0.1 g of the same feed sub-sample. All measurements for each feed sample were completed in a single day.
- *Between-day measurements.—*For between-day measurements on three separate days, each set of replicates (*n* = 18) was from a single 5 g feed test portion with each replicate again being 0.1 g of the same sub-sample. Replicates on each of the 3 days were on three different 5 g test portions which were separately spiked with the same concentrations of mycotoxins.
- *Determination of LODs and LOQs.—*Initial estimates of LODs and LOQs were made visually from the ion chromatograms as to the quantities of individual mycotoxins that should be detectable with S/N of 3:1 and 9:1, respectively. The estimates were then tested by spiking the matrices with the appropriate quantities from which LOD and LOQ values were confirmed. It was only possible to experimentally demonstrate achievable LODs and LOQs for individual mycotoxins which were not found to be naturally present in respective test feed materials. Where there was background contamination, the noise was measured close to the peak of interest and the LOD and LOQs estimated but not confirmed by subsequent spiking.
- *Method accuracy.—*In the absence of suitable certified reference materials, surplus test materials used in proficiency testing (FAPAS) were analyzed. The determined values for aflatoxins, DON, ZON, and T-2 and HT-2 toxins in two different cereal-based feed samples were compared with assigned values provided by the test material suppliers.

## Results and Discussion

### Method Development and Optimization

The loading, washing and elution conditions for employing IACs for cleanup of mycotoxins from a diverse range of matrices using columns containing antibodies for single mycotoxins are well established (29). These columns for each of the 11 mycotoxins individually have been employed in methods adopted as official methods for foodstuffs and feed materials by both AOAC and CEN after extensive interlaboratory validation (29). Additionally, although not subjected in inter-laboratory validation, columns for multi-mycotoxins, for example for three toxins such as DON, ZON, and T-2/HT2 toxins (30). and combinations of IACs in tandem (31, 32) have demonstrated the viability of multi-mycotoxin cleanup using IACs. One significant challenge for any multi-mycotoxin method is in optimizing extraction from an animal feed matrix for mycotoxins of different polarities ranging from OTA to T-2 toxin which necessitates some compromises. Of the various published multi-mycotoxin methods ACN–H_2_O–HCOOH (9, 15, 20, 22), ACN–H_2_O–acetic acid (MeCOOH) (11, 18), acidified MeOH (10), and MeOH–H_2_O (16, 17, 33) in various proportions and with differences in acidification have all been employed. Of these MeOH–H_2_O mixtures have mostly only been employed for IAC cleanup, whilst the other extraction mixtures have been employed more widely for “dilute and shoot” and modified QuEChERS cleanup.

In preliminary method development work, it was found that to achieve good recoveries of OTA, acidified extraction mixtures were needed and ACN–H_2_O–HCOOH (79 + 20 + 1) which has been successfully employed by others (9, 15, 20, 22) was adopted as being optimum. It was also found that during the IAC step the use of H_2_O to wash the column caused some breakthrough of OTA. This problem was overcome by replacing H_2_O with a wash solution of 20 mM ammonium acetate.

### Specificity of MRM for Each Feed/Mycotoxin Combination

**Figure 1.**
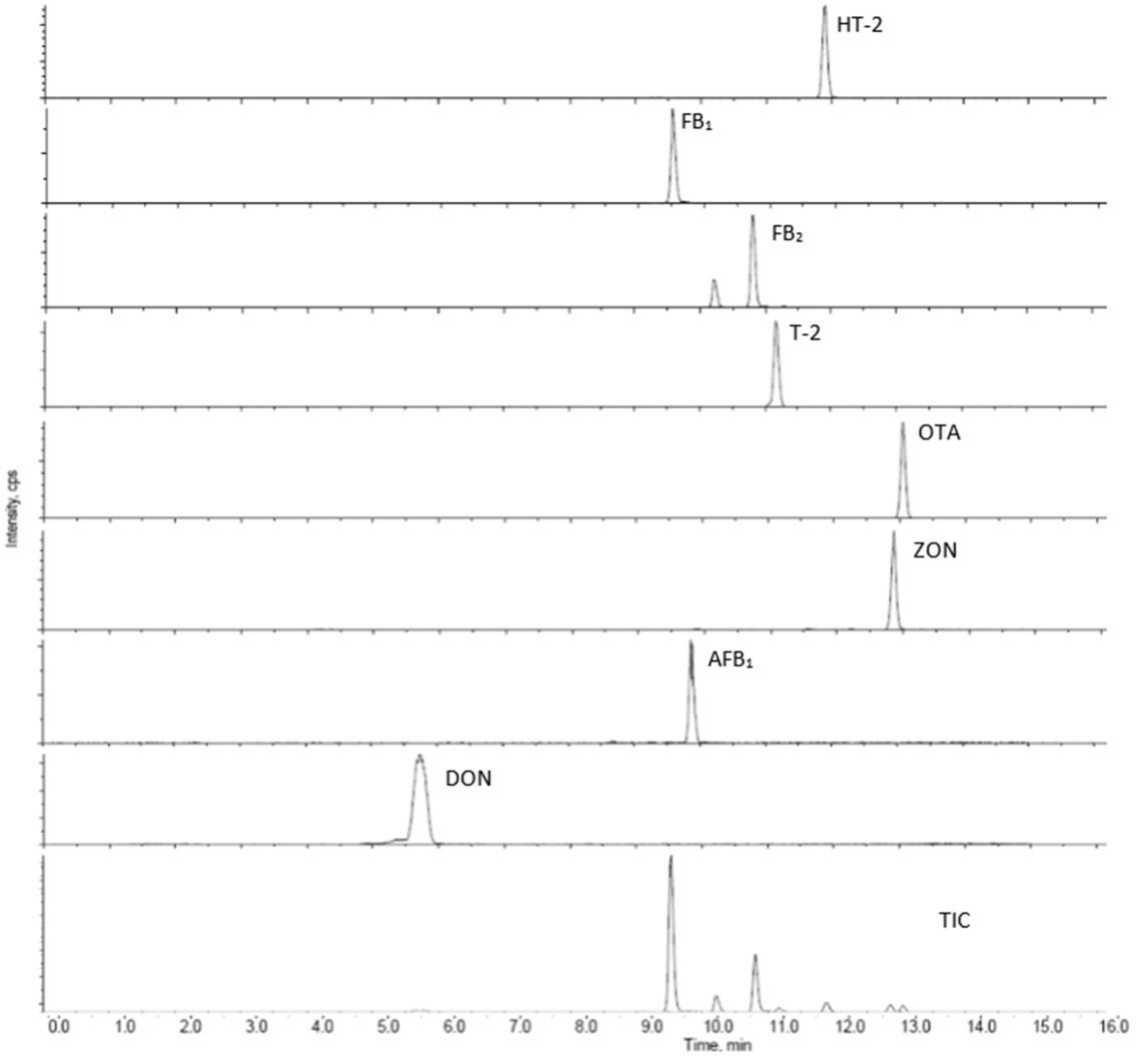
MRM ion chromatograms for DDGS sample containing 2459 µg/kg DON (*m/z* 297 →249), 17 µg/kg AFB1 (*m/z* 313 →285), 271 µg/kg ZON (*m/z* 319 →283), 50 µg/kg OTA (*m/z* 404 →239), 125 µg/kg T-2 toxin (*m/z* 484 →305), 2260 µg/kg FB2 (*m/z* 706 →366), 6273 FB1 (*m/z* 772 →334), 125 µg/kg HT2-toxin (*m/z* 442 →263). TIC – Total ion chromatogram.

The peak shapes of the ion chromatograms for each mycotoxin in each of the three feed samples were visually inspected and, in all cases, showed no evidence of shoulders or background interferences. DON typically had a broader peak than the other toxins despite early elution at 5.7 min. A typical ion chromatogram for DDGS containing AFB_1_, DON, FB_1_, FB_2_, HT-2, T-2, and ZON present as natural mycotoxin contaminants and spiked with OTA is shown in Figure 1. When no cleanup or alternatives to IAC are employed, there is invariably some background interference visually evident. This was exemplified by the analysis of HT-2 toxin and DON in pig feed where, when there was no sample cleanup, the MRM transitions could not be integrated because of background noise (29). In contrast, the same MRM transition ion peaks in extracts after IAC cleanup were essentially indistinguishable from that of standards (29).

A second check on specificity (30) was undertaken comparing ion ratios for individual mycotoxins in contaminated feed samples with the mean ion ratios for mycotoxin standards in solution. After feed samples had been through the multi-mycotoxin IAC cleanup there was good agreement of ion ratios with standards within a tolerance of ±20%.

A check was also made for possible matrix effects by comparing a calibration series for each standard in 50% MeOH and in blank sample extract. For all eleven mycotoxins and three feed samples tested, area response for analytes in both 50% MeOH and feed extract were comparable. This could only be tested for those mycotoxins essentially absent as natural contaminants from feed samples, but overall provided evidence for all the mycotoxins in one or more feed sample. Calibration curves for standards in MeOH compared with standards in IAC feed extract were more-or-less superimposable showing no evidence of ion-suppression or enhancement matrix effects. This confirms that solvent standards can be used with the multi mycotoxin IACs in place of expensive isotopic standard and negates the need to use matrix matched standards to correct for such matrix effects.

### Spiking of Feed Samples to Establish Mycotoxin Recoveries

Target levels for AFB_1_ were chosen to cover the range of EU regulatory limits (7) and for OTA, DON, sum of FB_1_ + FB_2_, sum of HT-2 + T-2 toxins and ZON to cover the range of EU guidance values (8). The low, medium, and high levels selected are shown in Table 5 with the low level being 50% of the lowest limit for the most sensitive animal species, medium being an intermediate level, and high approaching the uppermost guidance values. For T-2 and HT-2 toxins, the high spiking level was selected as 250 μg/kg, being the indicative maximum level for compound feed. For DON and the sum of FB_1_ + FB_2_, guidance values of 12 000 μg/kg and 60 000 μg/kg, respectively, for maize and maize products were excluded from the spiking ranges selected, as they would require further dilution prior to loading to avoid exceeding the sample capacity of the IAC.

**Table 5.** Target spiking levels (μg/kg) for animal feed samples

Code	Mycotoxin spike levels, μg/kg										
	AFB_1_	AFB_2_	AFG_1_	AFG_2_	OTA	DON	FB_1_	FB_2_	HT-2	T-2	ZON
Low	2.5	2.5	2.5	2.5	25	450	1667	833	62.5	62.5	50
Medium	5	5	5	5	50	900	3333	1667	125	125	100
High	20	20	20	20	100	5000	6666	3333	250	250	500

Analysis of the three feed samples selected for the method validation indicated that all three contained some background mycotoxin natural contamination as shown in Table 6. DDGS contained significant levels of AFB_1_, DON, FB_1_ + FB_2_, and ZON, and pig feed contained significant levels of HT-2 and T-2 toxins precluding spiking with these toxins. Poultry feed contained 16 μg/kg DON, 186 μg/kg total fumonisins, and 7.9 μg/kg total HT-2 + T-2 toxins however, as these only represented 3.6, 7.5, and 6.3% of the lowest proposed spike levels and would not have a significant impact, they were corrected for recovery measurements. Similarly, pig feed contained fumonisins and ZON representing only, respectively, 6.3 and 20.4% of the lowest proposed spike levels, and spiking was carried out by making appropriate recovery corrections. For DDGS spiking was only carried out for AFB_2_, AFG_1_, AFG_2_, OTA, and the sum of HT-2 and T-2 toxins where for the latter a 7.5% natural contamination correction was made to spiking recoveries. Overall, it was concluded that having both spiked and naturally contaminated feed samples enabled a balanced picture to be provided by the method validation.

**Table 6.** Mean natural contamination levels of mycotoxins (μg/kg) in feed samples (*n* = 6)

Code	Mycotoxin levels, μg/kg										
	AFB_1_	AFB_2_	AFG_1_	AFG_2_	OTA	DON	FB_1_	FB_2_	HT-2	T-2	ZON
DDGS	17	1	<0.2	<0.3	<0.2	2459	6273	2260	6	3	271
Pig feed	<0.2	<0.4	<0.2	<0.3	<0.2	<10	116	42	31	8	10
Poultry feed	<0.2	<0.4	<0.2	<0.3	<0.2	16	133	53	6	1	<2

### Within-Day Recovery and Precision Data

The within-day recovery data for spiked samples is shown in Table 7. For AFB_1_ for pig and poultry feed, recoveries ranged from 90–103%, but for DDGS with a 17 μg/kg AFB_1_ background level being three times the lowest regulated limit, spiking was precluded. However, for DDGS the average recovery for the sum of AFB_2_, AFG_1_, and AFG_2_ was 90.4% indicating good recoveries overall for total aflatoxins. For OTA, recoveries ranged from 101 to 114% for all three feed samples, whilst for pig feed, recoveries for DON, total fumonisins, and ZON averaged 96, 75 and 96% respectively. For poultry feed recoveries for DON, total fumonisins, total HT-2 + T-2, and ZON averaged 119, 90, 100, and 105% respectively. For DDGS, recoveries for the sum of HT-2 and T-2 toxins averaged 118% across the three spike levels.

**Table 7.** Average recoveries, %, (*n* = 6) for within-day analysis of spiked samples^a^

Feed	Level	AFB_1_	AFB_2_	AFG_1_	AFG_2_	OTA	DON	FB_1_	FB_2_	HT-2	T-2	ZON
DDGS	Low	—	92^b^	83	98	106	—	—	—	114^b^	117^b^	—
	Medium	—	106^b^	80	98	114	—	—	—	122^b^	126^b^	—
	High	—	91^b^	79	87	101	—	—	—	109^b^	118^b^	—
Pig feed	Low	90	74	83	82	101	103	66^b^	79^b^	—	—	94^b^
	Medium	95	92	91	85	109	106	77^b^	83^b^	—	—	99^b^
	High	90	91	90	84	102	80	71^b^	75^b^	—	—	94^b^
Poultry feed	Low	103	111	97	94	108	126^b^	84^b^	89^b^	102^b^	97^b^	105
	Medium	101	129	99	103	111	136^b^	89^b^	94^b^	102^b^	104^b^	109
	High	99	114	94	93	110	96^b^	93^b^	92^b^	98^b^	100^b^	100

a Natural contamination levels which precluded spiking indicated by — so no recovery data generated.

b Results with background contamination subtracted from measured values.

Although general guidance indicates that recoveries should be in the range of 70–110% (34) a range of 50–120% can be deemed acceptable for a limited number of analytes known as critical when using multi-analyte methods, under the provision that the standard deviation from the experiments is consistent and comparing with those obtained for naturally contaminated materials (34).

The within-day repeatability data shown as RSDs are presented in Table 8. The data (*n *=* *6) is shown both for those mycotoxins where spiking was undertaken at three spike levels as well as for mycotoxins where there was natural contamination at levels precluding spiking. Overall, the within-day RSDs ranged from 0.7% for ZON in pig feed up to 11.7% for AFG_2_, again in pig feed, and overall across spiking levels and feeds averaged 4.8%. EU guidance for method performance for human foodstuffs (35) albeit not specifically for animal feed sets requirements for relative standard deviation (RSD_r_) of <20% for OTA, DON, FB_1_, and FB_2_, <25% for ZON, and <30% for HT-2 and T-2 toxins. The values for RSDs shown in Table 6 are all well within these method performance criteria demonstrating the method is satisfactory for use for official methods purposes.

**Table 8.** Repeatability (RSD, %) for analysis of spiked and naturally contaminated mycotoxins in feed samples^a^

Feed	Level	AFB_1_	AFB_2_	AFG_1_	AFG_2_	OTA	DON	FB_1_	FB_2_	HT-2	T-2	ZON
DDGS	Low	5.5	9.7	7.3	9.5	2.6	2.1	5.1	4.9	3.7	3.7	2.6
	Medium	8.8	6.1	7.8	10.5	2.5	3.5	6.1	7.5	2.5	3.2	4.3
	High	4.2	1.8	2.8	3.7	3.1	2.0	4.5	5.1	2.9	3.6	3.3
Pig feed	Low	5.6	9.0	5.8	11.7	5.8	3.7	6.2	3.2	4.0	8.1	6.5
	Medium	4.8	7.5	6.5	6.9	3.8	5.3	6.2	5.3	6.6	10.3	2.9
	High	5.7	5.5	4.3	5.7	4.3	1.7	5.1	3.6	4.8	8.8	0.7
Poultry feed	Low	6.7	9.5	6.4	4.3	3.5	1.6	3.5	3.4	4.5	1.4	2.5
	Medium	8.7	4.8	5.0	5.9	3.8	1.9	3.1	4.3	2.1	2.6	3.1
	High	3.9	6.3	2.0	3.1	4.5	4.0	5.5	3.8	3.8	2.1	0.8

a Repeatability based on *n* = 6 for both spiked and naturally contaminated mycotoxins in feed samples.

### Between-Day Recovery and Precision Data

The between-day recovery data for spiked samples is shown in Table 9. For AFB_1_, for pig and poultry feed, recoveries ranged from 91–120%. For DDGS, the average recovery for the sum of AFB_2_, AFG_1_, and AFG_2_ was 90.6% indicating good recoveries overall for total aflatoxins. For OTA, recoveries ranged from 100–108% for all three feed samples, whilst for pig feed, recoveries for DON, total fumonisins, and ZON averaged 100, 78, and 101%, respectively. For poultry feed, recoveries for DON, total fumonisins, total HT-2 + T-2, and ZON averaged 114, 88, 95, and 103%, respectively. For DDGS, recoveries for the sum of HT-2 and T-2 toxins averaged 113% across the three spike levels. The recoveries for spiking were broadly comparable for within-day and between-day measurements and deemed acceptable.

**Table 9.** Average recoveries, %, (*n* = 6) for between-day analysis of spiked samples^a^

Feed	Level	AFB_1_	AFB_2_	AFG_1_	AFG_2_	OTA	DON	FB_1_	FB_2_	HT-2	T-2	ZON
DDGS	Low	—	85^b^	84	88	107	—	—	—	111^b^	111^b^	—
	Medium	—	111^b^	84	90	108	—	—	—	113^b^	114^b^	—
	High	—	104^b^	78	91	103	—	—	—	117^b^	116^b^	—
Pig feed	Low	100	109	94	96	105	109	73^b^	84^b^	—	—	105^b^
	Medium	96	107	93	91	103	110	75^b^	82^b^	—	—	99^b^
	High	91	105	88	87	100	80	76^b^	80^b^	—	—	98^b^
Poultry feed	Low	103	113	98	102	106	120^b^	81^b^	87^b^	96^b^	94^b^	103
	Medium	99	120	97	101	105	128^b^	84^b^	96^b^	96^b^	94^b^	103
	High	97	116	93	94	103	94^b^	90^b^	88^b^	97^b^	96^b^	102

a Natural contamination levels which precluded spiking indicated by — so no recovery data generated.

b Results with background contamination subtracted from measured values.

The between-day repeatability data shown as RSDs is presented in Table 10. The data (*n *=* *18) is shown both for those mycotoxins where spiking was undertaken at three spike levels as well as for mycotoxins where there was natural contamination at levels precluding spiking. The between-day RSDs ranged from 3.2% for HT-2 toxin in DDGS up to 39.4% for T-2 toxin in pig feed. Overall, across spiking levels and feeds RSDs averaged 9.3% and both the average and individual RSDs were significantly higher for between-day than within-day measurements. However, an RSD_r_ of <20% for OTA, DON, FB1, FB2, and <25% for ZON was achieved although three of the six measurements were around the maximum target of 30% for HT-2 and T-2 toxins in pig feed. As the between-day sub-samples were spiked on each of the three separate days it is possible that some imprecision in spiking is reflected in comparison with the within-day RSDs.

**Table 10.** Repeatability (RSD, %) for between-day analysis of spiked and naturally contaminated mycotoxins in feed samples^a^

Feed	Level	AFB_1_	AFB_2_	AFG_1_	AFG_2_	OTA	DON	FB_1_	FB_2_	HT-2	T-2	ZON
DDGS	Low	8.8	15.8	5.1	7.3	3.4	3.3	5.1	7.4	5.1	5.0	3.5
	Medium	20.3	15.8	14.1	12.7	6.8	5.5	6.8	5.8	7.9	8.9	4.5
	High	12.0	12.4	7.0	7.3	4.3	5.8	4.3	12.1	3.2	3.2	6.3
Pig feed	Low	13.2	27.7	12.9	14.0	5.3	13.1	8.9	7.6	15.9	39.4	14.0
	Medium	7.6	17.7	10.0	13.2	6.4	6.7	5.5	3.9	17.9	13.2	5.5
	High	10.0	15.8	8.9	9.8	6.0	10.3	7.2	6.6	31.2	29.9	6.9
Poultry feed	Low	7.5	11.0	7.7	9.3	5.3	10.6	4.2	4.1	7.4	4.3	3.8
	Medium	9.7	9.5	6.6	6.9	6.8	10.1	6.1	8.2	6.8	13.1	7.0
	High	9.0	7.8	6.6	9.0	9.5	5.2	7.2	6.7	6.9	7.1	5.0
CEN^b^	Lowest	0.5	n/a	n/a	n/a	4.1	4.0	4.4	4.8	3.6	5.1	3.9
	Highest	24.1	n/a	n/a	n/a	12.1	6.9	7.8	9.3	84.6	10.1	37.4

a Repeatability based on *n* = 18 for both spiked and naturally contaminated mycotoxins in feed samples.

b Repeatability data taken from CEN multi-mycotoxin standard (20).

Like-for-like comparisons of method performance between different studies is difficult as matrices and spiking levels differ significantly. However, in Table 10 the lowest and highest percentage RSD_r_ values reported from a CEN multi-mycotoxin interlaboratory study (20) are shown. The between-day RSDs reported in this paper broadly fall within the ranges for the CEN study for AFB_1_, OTA, DON, FB_1_, FB_2_, and ZON. However, both studies show anomalously high RSDs in some, but not all, instances for HT-2 and T-2 toxins which indicates greater variability regardless of the method used when analyzing these two toxins.

### LODs and LOQs

The LODs and LOQs for the individual mycotoxins in each of the feed matrices are shown in Table 11. These values are substantially below the lowest EU guidance values for the most sensitive animal species also indicated in Table 11. Only for AFB_1_ which has the lowest regulatory limit of 5.0 μg/kg, is the method LOQ significant and it is still approximately eight times below the regulatory limit. Table 11 also indicates the LOQs that need to be demonstrated in the application of the CEN multi-mycotoxin standard (20) which are easily satisfied except for FB_2_, although a 500 μg/kg requirement for total FB_1_ + FB_2_ is satisfied. Overall, it can be concluded that the LODs and LOQs are lower than the levels required with respect to guidance values demonstrating the suitability of the proposed method.

**Table 11.** LODs and LOQs estimated and demonstrated by spiking (μg/kg) into feed samples

Feed sample	LODs, μg/kg										
	AFB_1_	AFB_2_	AFG_1_	AFG_2_	OTA	DON	FB_1_	FB_2_	HT-2	T-2	ZON
DDGS	0.2	0.4	0.2	0.3	0.2	21.1	125	104	1.0	0.2	2.0
Pig feed	0.2	0.4	0.2	0.3	0.2	10.5	0.9	1.6	1.0	0.2	2.0
Poultry feed	0.2	0.4	0.2	0.3	0.2	12.6	0.8	0.4	1.0	0.2	2.0

	LOQs, μg/kg										

DDGS	0.6	1.2	0.6	0.9	0.5	63.3	376	313	3.0	0.7	6.0
Pig feed	0.6	1.2	0.6	0.9	0.5	31.6	2.8	4.7	3.0	0.7	6.0
Poultry feed	0.6	1.2	0.6	0.9	0.5	38.0	2.3	1.2	3.1	0.7	6.0
CEN^a^	<2.0	NA^b^	NA	NA	<10	<100	<375	<125	<10	<10	<20
EU limits	5.0	NA	NA	NA	50	900	5000		250		100

a Miminum requirements specified in CEN multi-mycotoxin standard (20).^b^NA = Not applicable.

### Method Accuracy

The accuracy of the method was tested by analysis of two cereal-based animal feed samples both naturally contaminated with mycotoxins and which had been used as proficiency test samples. The results of this analysis are shown in Table 12. For feed sample 1 assigned values were only provided for the four individual aflatoxins for which the measured values showed close agreement with presumed *z*-scores of −0.1, −0.1, −0.5, and −0.8 for AFB_1_, AFB_2_, AFG_1_, and AFG_2_, respectively where a score of ±2.0 is deemed satisfactory. Additionally, using the multi-mycotoxin method 11.2 μg/kg ZON was found although there was no assigned value for this mycotoxin. For feed sample 2, assigned values were given for DON, HT-2, T-2 toxins, and ZON and measured values show good agreement with presumed *z*-scores of 0.5, 1.3, 0.8, and 1.1, respectively, again well within the ±2.0 range for satisfactory results. At the time of this method validation work there were no surplus feed test materials containing assigned values for fumonisins or OTA so the accuracy of measurement of these toxins could not be established.

**Table 12.** Measured levels of mycotoxins in two FAPAS QC samples compared to assigned values

QC samples	Mycotoxin levels, µg/kg								
	AFB_1_	AFB_2_	AFG_1_	AFG_2_	OTA	DON	HT-2	T-2	ZON
Feed sample 1									
Found values	8.5	4.2	3.4	1.3	<0.2	<10	<1.0	<0.2	11.2
Assigned values	8.8	4.4	3.9	1.6	NA^a^	NA	NA	NA	NA
Feed sample 2									
Found values	<0.2	<0.4	<0.2	<0.3	<0.2	967	399	245	182
Assigned values	NA	NA	NA	NA	NA	900	310	212	146

a NA = Not applicable as assigned values only reported for aflatoxins for sample 1 and only reported for DON, HT-2, T-2, and ZON for sample 2.

## Conclusions

In this single-laboratory method validation study, a multi-mycotoxin IAC method employing LC–MS/MS has been shown for three complex animal feed samples to give acceptable recoveries and precision for 11 mycotoxins of concern in animal feed. The use of IAC cleanup has benefits compared to alternative approaches as the rigor of the cleanup avoids the need to compensate for co-extractives by matrix-matched calibration, stable isotope internal standards, and/or careful selection of chromatographic and MS/MS conditions. The use of IAC cleanup opens the way to fully automated systems as has already been demonstrated for aflatoxin analysis using reuseable cartridges containing monoclonal antibodies to aflatoxins coupled to a pressure resistant polymer (36, 37). This single-laboratory study provides strong evidence of good method performance to justify a future full interlaboratory study of the proposed method.

## CRediT Author Statement

**Naomi Mackay:** Methodology, Formal analysis, Validation, Data curation, Writing—review & editing. **Elaine Marley:** Methodology, Conceptualization. **Dave Leeman:** Methodology, Conceptualization. **Cezary Poplawski:** Methodology, Conceptualization. **Carol Donnelly:** Conceptualization, Supervision, Writing—review & editing.

## Funding

This research was fully funded by R-Biopharm Rhone Ltd, Block 10, Todd Campus, West of Scotland Science Park, Acre Rd, Glasgow G20 0XA, United Kingdom.

## Conflict of Interest

All authors declare no conflict of interest.

## Supplemental Information

Supplemental information is available on the *J. AOAC Int.* website.

## Supplementary Material

qsac035_Supplementary_Data
