# Supplementary material for: Analysis of Aflatoxins, Fumonisins, Deoxynivalenol, Ochratoxin A, Zearalenone, HT-2, and T-2 Toxins in Animal Feed by LC–MS/MS Using Cleanup with a Multi-Antibody Immunoaffinity Column
Source: J AOAC Int. 2022 Mar 8;105(5):1330–40. doi: 10.1093/jaoacint/qsac035 (PMC9446684; doi:10.1093/jaoacint/qsac035)
Supplement: qsac035_Supplementary_Data [file qsac035_supplementary_data.docx]

**Table S1. Volumes of standard solutions used for spiking DDGS**

Low level

5 g of control sample was spiked with the following volumes of each standard solution:

| **Mycotoxin** | **Target level (ng/g)** | **Standard soln conc. (ng/mL)** | **Volume added (µL)** |
| --- | --- | --- | --- |
| AFB2 | 2.5 | 100 | 125 |
| AFG1 | 2.5 | 100 | 125 |
| AFG2 | 2.5 | 100 | 125 |
| OTA | 25.0 | 1000 | 125 |
| T-2 + HT-2 | 125.0 | 4000 | 156 |

Medium level

5 g of control sample was spiked with the following volumes of each standard solution

| **Mycotoxin** | **Target level (ng/g)** | **Standard soln conc. (ng/mL)** | **Volume added (µL)** |
| --- | --- | --- | --- |
| AFB2 | 5.0 | 1000 | 25 |
| AFG1 | 5.0 | 1000 | 25 |
| AFG2 | 5.0 | 1000 | 25 |
| OTA | 50.0 | 1000 | 250 |
| T-2 + HT-2 | 250.0 | 40,000 | 31 |

High Level
5 g of control sample was spiked with the following volumes of each standard solution:

| **Mycotoxin** | **Target level (ng/g)** | **Standard soln conc. (ng/mL)** | **Volume added (µL)** |
| --- | --- | --- | --- |
| AFB2 | 20.0 | 1000 | 100 |
| AFG1 | 20.0 | 1000 | 100 |
| AFG2 | 20.0 | 1000 | 100 |
| OTA | 100.0 | 1000 | 500 |
| T-2 + HT-2 | 500.0 | 40,000 | 63 |

**Table S2. Volumes of standard solutions used for spiking pig feed**

Low level

5 g of control sample was spiked with the following volumes of each standard solution:

| **Mycotoxin** | **Target level (ng/g)** | **Standard soln conc. (ng/mL)** | **Volume added (µL)** |
| --- | --- | --- | --- |
| Total AFL | 10 | 1000 | 50 |
| OTA | 25 | 1000 | 125 |
| FB1 + FB2 | 2500 | 100,000 | 125 |
| DON | 450 | 100,000 | 23 |
| ZON | 50 | 5000 | 50 |

Medium level

5 g of control sample was spiked with the following volumes of each standard solution

| **Mycotoxin** | **Target level (ng/g)** | **Standard soln conc. (ng/mL)** | **Volume added (µL)** |
| --- | --- | --- | --- |
| Total AFL | 20 | 1000 | 100 |
| OTA | 50 | 1000 | 250 |
| FB1 + FB2 | 5000 | 100,000 | 250 |
| DON | 900 | 100,000 | 45 |
| ZON | 100 | 50,000 | 10 |

High Level
5 g of control sample was spiked with the following volumes of each standard solution:

| **Mycotoxin** | **Target level (ng/g)** | **Standard soln conc. (ng/mL)** | **Volume added (µL)** |
| --- | --- | --- | --- |
| Total AFL | 80 | 1000 | 400 |
| OTA | 100 | 1000 | 500 |
| FB1 + FB2 | 10,000 | 100,000 | 500 |
| DON | 5000 | 100,000 | 250 |
| ZON | 500 | 50,000 | 50 |

**Table S3. Volumes of standard solutions used for spiking poultry feed**

Low level

5 g of control sample was spiked with the following volumes of each standard solution:

| **Mycotoxin** | **Target level (ng/g)** | **Standard soln conc. (ng/mL)** | **Volume added (µL)** |
| --- | --- | --- | --- |
| Total AFL | 10 | 1000 | 50 |
| OTA | 25 | 1000 | 125 |
| FB1 + FB2 | 2500 | 100,000 | 125 |
| DON | 450 | 100,000 | 23 |
| ZON | 50 | 5000 | 50 |
| HT-2 + T-2 | 125 | 4000 | 126 |

Medium level

5 g of control sample was spiked with the following volumes of each standard solution

| **Mycotoxin** | **Target level (ng/g)** | **Standard soln conc. (ng/mL)** | **Volume added (µL)** |
| --- | --- | --- | --- |
| Total AFL | 20 | 1000 | 100 |
| OTA | 50 | 1000 | 250 |
| FB1 + FB2 | 5000 | 100,000 | 250 |
| DON | 900 | 100,000 | 45 |
| ZON | 100 | 5000 | 100 |
| HT-2+T-2 | 250 | 40,000 | 31 |

High Level
5 g of control sample was spiked with the following volumes of each standard solution:

| **Mycotoxin** | **Target level (ng/g)** | **Standard soln conc. (ng/mL)** | **Volume added (µL)** |
| --- | --- | --- | --- |
| Total AFL | 80 | 1000 | 400 |
| OTA | 100 | 1000 | 500 |
| FB1 + FB2 | 10,000 | 100,000 | 500 |
| DON | 5000 | 100,000 | 250 |
| ZON | 500 | 50,000 | 50 |
| HT-2+T-2 | 500 | 40,000 | 63 |
